# 22^nd^ AMN Congress in Bangkok, Thailand – Interview with General Siraruj Sakoolnamarka

**DOI:** 10.25122/jml-2025-1008

**Published:** 2025-10

**Authors:** Alexandra Gherman, Stefana-Andrada Dobran

**Affiliations:** 1 RoNeuro Institute for Neurological Research and Diagnostic, Cluj-Napoca, Romania; 2 Sociology Department, Babes-Bolyai University, Cluj-Napoca, Romania

**Interviewee: General Siraruj Sakoolnamarka**

**Interviewer: Alexandra Gherman**

General Siraruj Sakoolnamarka served as Co-Congress Chair of the 22^nd^ Congress of the Academy for Multidisciplinary Neurotraumatology (AMN) and the Co-Organizer of the NTSC Extended AMN Intensives Thailand Edition.

A distinguished specialist, he serves as a Consultant Neurosurgeon at Bangkok International Hospital and holds the position of Senior Consultant Neurosurgeon and Head of the Stroke Center at Phramongkutklao Army Hospital. His influence in the field is reflected through his several leadership roles. Currently, he is the Chairman of the Thai Neurovascular Board Exam Committee (2025-2027) and Consultant for the Thai Neurological Surgery Board Exam Committee (2019-now). Previously, he held the position of president for both the Royal College of Neurosurgical Surgeons of Thailand (2017-2019) and of the Thai Association of Neurovascular Surgeons (2023-2025).

His clinical expertise covers epilepsy surgery, pediatric neurosurgery, and intraoperative neuromonitoring.


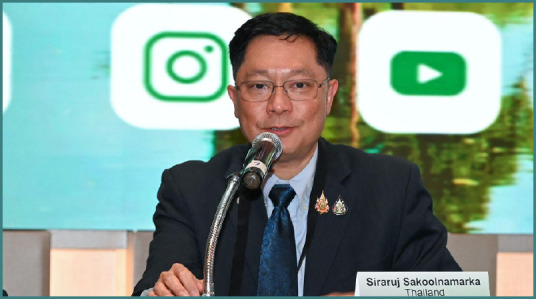


**A.G.: Dear General Siraruj Sakoolnamarka, thank you so much for being here. What is your perspective on AMN as a global player in the practice and science fields of TBI?**

Sir.S: My perspective on AMN is that AMN is not just a scientific organization, it's a powerful platform for change. It gives us a unique chance to connect to global experts and explore the latest cutting-edge research and take part in a discussion where many of us do not have access to. What makes AMN truly special is its ability to bring hope and real solutions, not just through science, but through practical training, international collaborations, and also the shared goal of improving outcomes in neurotrauma. It also empowers professionals from every stage of patient care ‒ emergency frontier to long-term rehabilitation – by providing tools to improve practice and influence health policy and contribute global knowledge. All together, the AMN is helping write the standard of neurotraumatology – exactly what is needed most around the world.


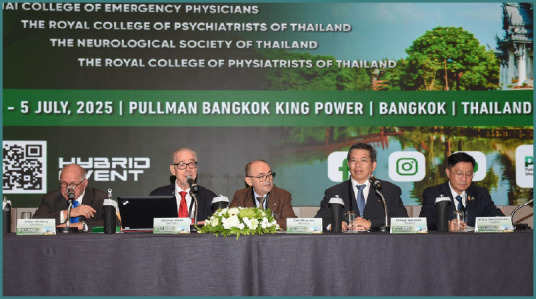


**A.G.: The 2025 Congress was preceded by the first edition of the AMN Intensives Neurotrauma Simulation Center Thailand teaching course. As a faculty member, please share your opinion regarding this educational event**.

Sir.S: The AMN Committee approved the 2025 NTSC Extended AMN Intensives in Bangkok about a year ago. It's truly a great honor for us to host this precious program ‒ and I'm proud to be part of it, one that we believe we make a meaningful and lasting impact. From the beginning, my colleagues and I have been working closely to prepare for this event which we see as not just a meeting but a movement to elevate neurotrauma education and care.

First of all, we now have a new international standard simulation center right here in Bangkok. I must express my sincere gratitude to Professor Christian Matula, Professor Peter Lackner, and Professor Dafin Muresanu, for their invaluable guidance and support. I think it wouldn't have been possible without them. I’m thankful for their mentorship. Now we are confident and well prepared to meet the expectations of all our international participants.

Secondly, we embrace the core philosophy of the AMN: multidisciplinary collaborations. My team and I have come to appreciate just how crucial it is for professionals from different specialties to work together, sharing knowledge, reviewing each other's practice, building new protocols, and creating structural records for future research. This has transformed our approach from focusing only on short-term treatment to delivering long-term team-based care.

Thirdly, we are proud to share that a number of new research projects have emerged from this collaboration and present during this meeting. This reflects not just the academic effort but real progress in how we understand and treat neurotrauma. And finally, one of the most rewarding outcomes has been seeing younger generations, including medical students who joined this NTSC. They have had the opportunity to observe, learn practical skills, and engage with the professionals. We believe this early experience will inspire and shape their future career.

In conclusion, I truly hope the changes that we made - both in mindset and in practice - will continue to grow and lead to a sustainable and progressive future for neurotrauma care here in Thailand and beyond.


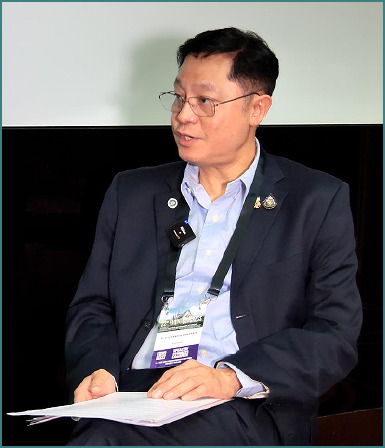


**A.G.: Congratulations on the Simulation Center! We were all there and we saw the great facilities. And I must say that I also saw you in action at the cadaveric workshops with a part of the participants. I just wanted to ask you, how did you feel about the participants, the young medical doctors, were they excited to be there, curious, anxious to learn? I believe it's very rewarding for you to teach and to see them all there**.


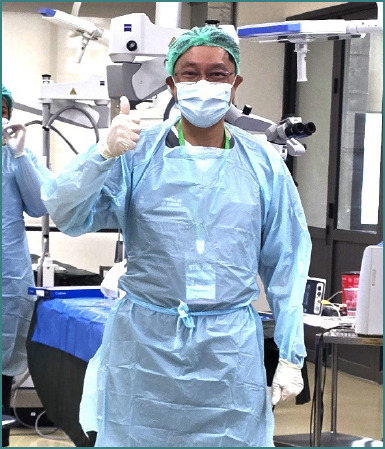


Sir.S: That is… a new experience for them. Usually, they will most of the time study in the textbook and now they can see the simulation environments and also get in touch with the professional. So, I think this is very helpful to share with them and lead them in the future.

**A.G.: As a neurosurgeon and co-chair of the event, what is your intake related to the 2025 AMN Congress and what would you foresee as future developments for the complex multidisciplinary domain in TBI?**

Sir.S: Yes, of course, I have two points. The first one is TBI or traumatic brain injury, it’s never just one person or one specialty - it takes a whole team working together from pre-hospital responders, emergency physicians, ICU doctors, neurologists, neurosurgeons, physiatrists, psychiatrists, and nurses. Everyone plays a vital role and I hope this program helps all of us see the importance of seamless, well-coordinated care across every stage of treatment. Secondly, our team is also working hard to build a stronger system. We are developing a national trauma registry, creating clinical guidelines, regularly attending workshops, and actively producing research to drive evidence-based care. These efforts are helping raise the standard of neurotrauma care across the country. And of course, we must never forget that prevention is better than cure. So, we need to take the lead in promoting public awareness - through social media communication, community outreach and nationwide campaigns - to help reduce the number of preventable injuries before they happen.


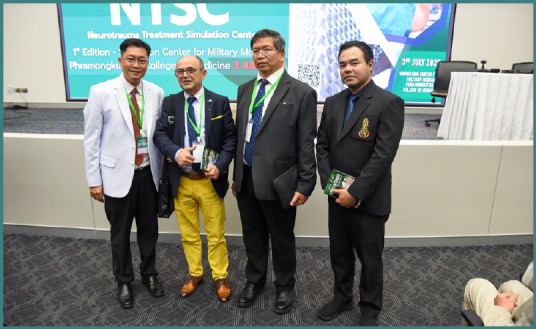


**A.G.: At a personal level, how would you envision your future collaboration within the AMN academic environment?**

Sir.S: Firstly, I really love the idea of the Focus Groups. I'm excited to set up one focus group in Thailand. We plan to bring together professionals from different fields who truly understand and support the AMN concept. After we come together to brainstorm, the team will design long-term projects and develop clear plans to implement them. And secondly, since we are already established as a stimulation-based learning center for neurotraumatology, we are fully ready to support the program and we would be more than honored if AMN chooses our center as a host in the future.

**A.G. Thank you so much! I think you are the first to embrace this initiative of the Focus Group. Actually, the first to announce embracing this initiative of the AMN Focus Groups and I wish you best of luck in all your endeavors!**

